# The *Opuntia streptacantha OpsHSP18* Gene Confers Salt and Osmotic Stress Tolerance in *Arabidopsis thaliana*

**DOI:** 10.3390/ijms130810154

**Published:** 2012-08-15

**Authors:** Silvia Salas-Muñoz, Gracia Gómez-Anduro, Pablo Delgado-Sánchez, Margarita Rodríguez-Kessler, Juan Francisco Jiménez-Bremont

**Affiliations:** 1 Division of Molecular Biology, Institute Potosino of Scientific and Technological Research, Camino a la Presa de San José 2055, P.O.B. 3-74, C.P. 78216, Tangamanga, San Luis Potosí, SLP, Mexico; E-Mail: silvia.salas@ipicyt.edu.mx; 2 Agriculture in Dry Land Areas, The Northwest Centre of Biological Research, Mar Bermejo No. 195, Col. Playa Palo de Santa Rita, P.O.B. 128, C.P. 23090, La Paz, BCS, Mexico; E-Mail: gatito_comiendo@hotmail.com; 3 Faculty of Agronomy, Universidad Autónoma de San Luis Potosí, Km. 14.5, Carretera San Luis Potosí-Matehuala, Soledad de Graciano Sánchez, P.O.B. 32, C.P. 78321, San Luis Potosí, SLP, Mexico; E-Mail: pablo.delgado@uaslp.mx; 4 Faculty of Sciences, Universidad Autónoma de San Luis Potosí, Salvador Nava s/n, C.P. 78290, Col Lomas, San Luis Potosí, SLP, Mexico; E-Mail: mrodriguez@fc.uaslp.mx

**Keywords:** abiotic stress, abscisic acid, *Arabidopsis thaliana*, germination, *Opuntia streptacantha*, small heat shock protein

## Abstract

Abiotic stress limits seed germination, plant growth, flowering and fruit quality, causing economic decrease. Small Heat Shock Proteins (sHSPs) are chaperons with roles in stress tolerance. Herein, we report the functional characterization of a cytosolic class CI sHSP (OpsHSP18) from *Opuntia streptacantha* during seed germination in *Arabidopsis thaliana* transgenic lines subjected to different stress and hormone treatments. The over-expression of the *OpsHSP18* gene in *A. thaliana* increased the seed germination rate under salt (NaCl) and osmotic (glucose and mannitol) stress, and in ABA treatments, compared with WT. On the other hand, the over-expression of the *OpsHSP18* gene enhanced tolerance to salt (150 mM NaCl) and osmotic (274 mM mannitol) stress in *Arabidopsis* seedlings treated during 14 and 21 days, respectively. These plants showed increased survival rates (52.00 and 73.33%, respectively) with respect to the WT (18.75 and 53.75%, respectively). Thus, our results show that *OpsHSP18* gene might have an important role in abiotic stress tolerance, in particular in seed germination and survival rate of Arabidopsis plants under unfavorable conditions.

## 1. Introduction

Abiotic stress such as drought and salinity impose serious limitations to plant growth, development and crop productivity [1]. In order to survive adverse conditions, plants have developed multiple strategies, including physiological, biochemical and molecular mechanisms to counteract damages caused by environmental conditions. At the molecular level, the expression of stress responsive genes has been classified into: the early- and the delayed-response genes. The first group of genes is induced very quickly and often transiently after stress perception, such as kinases and transcription factors. The delayed-response genes are activated more slowly and their expression is often sustained during the stress conditions, encoding stress proteins such as heat shock proteins (HSPs), late embryogenesis abundant (LEAs), aquaporins, enzymes for osmolyte metabolism, antioxidant proteins among others [2–5].

HSPs or molecular chaperones are produced in all organisms in response to elevated temperatures and other stress conditions; its induction is correlated with the acquisition of thermotolerance [6–8]. HSPs are involved in a variety of cellular processes including protein folding, assembly of oligomeric proteins, transport of proteins across membranes, stabilization of polypeptide strands and membranes, and prevention of protein inactivation [8–10]. In addition, HSPs participate in regulating the activation and function of target proteins, although themselves are not components of the target proteins [10]. These proteins are classified in plants into five classes according to their molecular weight: HSP100, HSP90, HSP70, HSP60, and small HSP (sHSP) [9,11,12].

sHSPs constitute a family of structurally diverse and abundant HSPs synthesized by plants, usually undetectable under normal conditions but they can be induced by stress conditions and developmental stimuli [13]. The monomeric molecular mass of sHSPs ranges from 12–42 kDa, but in their native state the majority of sHSPs form homo- and hetero-oligomers of 12 to 32 subunits [14], although there are also dimeric and tetrameric sHSPs [12,15]. sHSPs are defined by a core α-crystalline domain of about 100 amino acids that is conserved in all plant sHSPs. This domain is responsible for dimer formation (sHSP-sHSP), which is enriched in β-strands organized in a β-sheet sandwich, the basic structural unit of many sHSPs [16]. Also, this α-crystalline domain is flanked by an *N*-terminal region highly variable in size and sequence that has been suggested as a candidate site for substrate binding. On the other hand, the *C*-terminal region is short and flexible, and it has been proposed as the site of contact that stabilizes the oligomeric structure [12,14,17]. In the *Arabidopsis thaliana* genome, at least 19 open reading frames (ORFs) encoding sHSPs have been identified [18]. These proteins are grouped into six classes, based on their intracellular localization, three sHSP classes (CI, CII and CIII) are localized in the cytosol or nucleus, and other classes in plastids (class P), in the endoplasmic reticulum (class ER) and in the mitochondria (class M). In addition, a set of sHSP-like proteins have been described and grouped as: class CI related and class P related proteins [18].

Cactus pear (*Opuntia* spp.) is a perennial arborescent cactus widely distributed in the arid and semiarid regions of Mexico, where it plays a strategic role in subsistence agriculture. Cactus pear is edible as a fruit and vegetable, and is also used as forage crop and for medicinal purpose. *Opuntia* plants exhibit Crassulacean Acid Metabolism (CAM) photosynthesis, in which CO_2_ exchange occurs at night, when the water vapor pressure difference between the air and the transpiring surfaces is lower, resulting in high water use efficiency [19]. These plants present several extraordinary morphological adaptations to reduce water loss, for example: the globular or racquet-like structures known as cladodes, which store large amounts of water, the replacement of leaves by thorns, thick cuticle cladode, low stomatal frequency, and sunken stomata [20,21]. Because of these adaptations and its tolerance to drought, this plant can cope with a variety of environmental conditions [22,23].

Recently, our group reported a cDNA library of *Opuntia streptacantha* cladodes containing a collection of ESTs accumulated under abiotic stress [24]. Among the genes involved in stress response obtained in this library, a small heat shock protein (sHSP) was identified. In the present study, we have approached the functional characterization of the *OpsHSP18* gene, encoding an 18.34 kDa sHSP, by analyzing a series of *Arabidopsis thaliana* over-expressing lines *35S::OpsHSP18*. Results reveal that *35S::OpsHSP18* over-expressing lines are tolerant to salt and osmotic stress, suggesting that OpsHSP18 protein has a role in plant abiotic stress tolerance.

## 2. Results

### 2.1. The *OpsHSP18* Gene Encodes a Small Heat Shock Protein (sHSP)

A full length cDNA sequence (802 bp, Accession No. HO058719) encoding a small heat shock protein (OpsHSP18) from *Opuntia streptacantha* was isolated from a cDNA library previously generated under stress conditions [24]. The cDNA sequence comprises a 5′-UTR region of 95 bp, a 3′-UTR region of 221 bp, and an open reading frame (ORF) of 486 bp encoding a polypeptide of 161 amino acids with a predicted molecular mass of 18.34 kDa and an isoelectric point (pI) of 5.85 (Figure S1). In addition, a genomic fragment containing the *OpsHSP18* gene plus 272 bp of promoter region was obtained by inverse PCR method (Figure S1). A canonical TATA-box was found at −30 bp of the +1 transcription start site. No intron sequences were found within the *OpsHSP18* gene (Figure S1).

**Figure 1:**
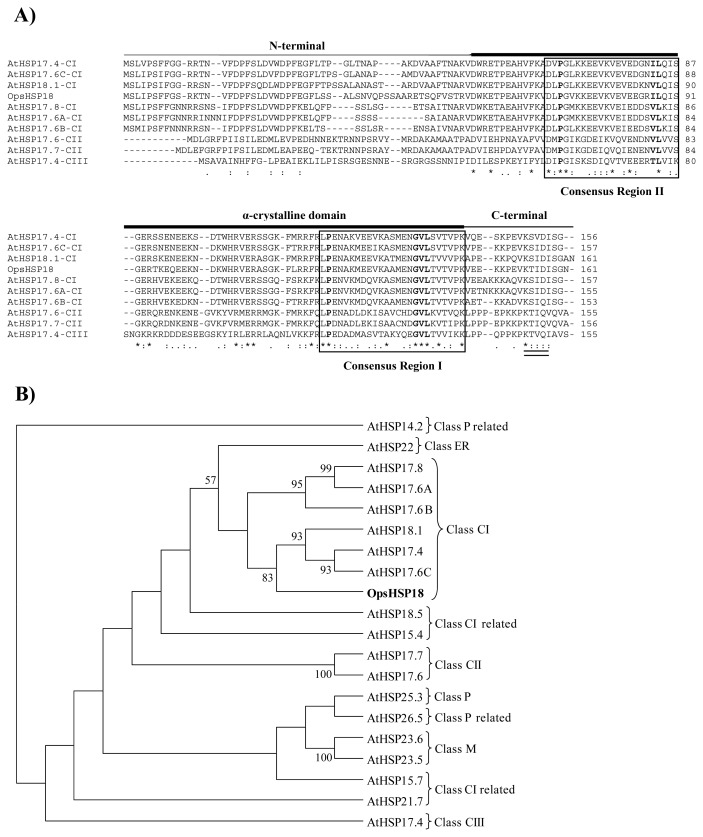
(**A**) Amino acid sequence alignment of OpsHSP18 and cytosolic class CI, CII and CIII proteins from *A. thaliana*. Characteristic domains are indicated with lines: *N*-terminal, α-crystalline and *C*-terminal domains. Two consensus regions I and II (boxed) and a conserved sequence motif, basic-X-Ile/Val-X-Ile/Val (double underlined). Identical residues (asterisk) and conserved amino acid substitutions (dots) are indicated. (**B**) Unrooted phylogenic tree of 19 small heat shock proteins of *A. thaliana* and the *O. streptacantha* OpsHSP18 protein. The phylogenetic tree was created by Neighbor-Joining method, PHYLIP 3.67 package [28]. Bootstrap support values out of 1000 pseudoreplicates of the data set, and are provided as percentages at the corresponding nodes when >50%. Proteins were grouped into six classes according to Scharf *et al*. [18]: class CI, CII and CIII (cytosolic/nucleus), class P (plastids), class ER (endoplasmic reticulum) and class M (mitochondria). sHSP-like proteins are indicated as: class CI related and class P related proteins.

The deduced amino acid sequence of the *O. streptacantha* OpsHSP18 protein and cytosolic class CI, CII, and CIII proteins from *A. thaliana* revealed characteristic features of plant sHSPs, such as typical α-crystalline domain (86 amino acids). This domain contains two consensus regions I and II (Figure 1A; boxed), separated by a hydrophilic region of variable length (Figure 1). The consensus regions present the Pro-X_(14)_-Gly-Val-Leu and Pro-X_(14)_-X-Val/Leu/Ile-Val/Leu/Ile motifs, in regions I and II (in bold), respectively. Both motifs are typical signatures of almost all plants sHSP (Figure 1A) [12,25,26]. Another conserved motif is basic-X-Ile/Val-X-Ile/Val, located in the *C*-terminal region and, suggested to be involved in the formation of oligomers of the plant sHSPs (Figure 1A; double underlined) [18,27].

A phylogenetic tree was constructed with the OpsHSP18 protein and 19 sequences of *A. thaliana* sHSP proteins (14.2 to 26.5 kDa; Figure 1B). According to the phylogenetic tree, the OpsHSP18 protein is clustered with the *A. thaliana* cytosolic class CI sHSPs, mainly with AtHSP17.4 (At3g46230), AtHSP17.6C (At1g53540) and AtHSP18.1 (At5g59720) proteins (Figure 1B). Amino acid sequence alignments of the OpsHSP18 protein with orthologous cytosolic class CI sHSPs of other plant species shows high sequence identity, especially in α-crystalline domain (Figure S2). The OpsHSP18 protein shows 79.60% identity with the *Citrullus lanatus* ClHSP18.1A, 79.50% with the *Eucalyptus grandis* EgHSP18, 79.00% with the *Populus trichocarpa* PtHSP17.8, 78.50% with the *Vitis vinifera* VvHSP18.2, 77.30% with the *Medicago sativa* MsHSP18.2, 77.30% with the *A. lyrata* AlHSP18, and 75.30% with the *A. thaliana* AtHSP17.6C proteins.

### 2.2. *OpsHSP18* Gene Is Involved in the Response to Abiotic Stress during Seed Germination

**Figure 2:**
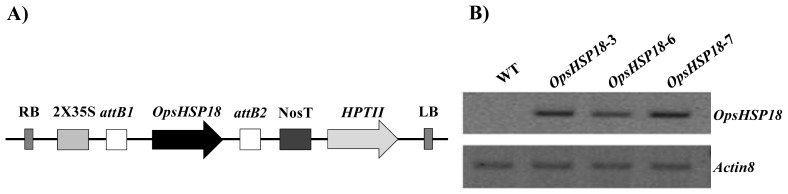
(**A**) Schematic diagram of the OpsHSP18 construction in pMDC32 binary vector. RB right border for T-DNA integration, 2 × 35S cauliflower mosaic virus (CaMV) promoter, *attB1* and *attB2* sites for recombination, OpsHSP18 (small heat shock protein 18) ORF from *Opuntia streptacantha*, NosT nopaline synthase (Nos) terminator region, *HPTII* hygromycin resistance gene, LB left border for T-DNA integration. (**B**) Detection of *OpsHSP18* transcript in *Arabidopsis thaliana 35S::OpsHSP18*-3, -6, and -7 over-expressing lines by semi-quantitative RT-PCR. Total RNA was isolated from 15 day-old *A. thaliana* seedlings. One microgram of total RNA was used for RT-PCR analysis. *A. thaliana Actin8* gene was used as loading control.

In order to elucidate the possible participation of *OpsHSP18* gene under stress conditions, *A. thaliana* over-expressing lines *35S::OpsHSP18*-3, *35S::OpsHSP18*-6, and *35S::OpsHSP18*-7, were generated (Figure 2A). The expression levels of *OpsHSP18* gene in Arabidopsis was determined by semi-quantitative RT-PCR, observing that all transgenic lines showed expression of the *OpsHSP18* gene, in comparison to control WT plants (Col-0), in which as expected, no expression was detected (Figure 2B).

**Figure 3:**
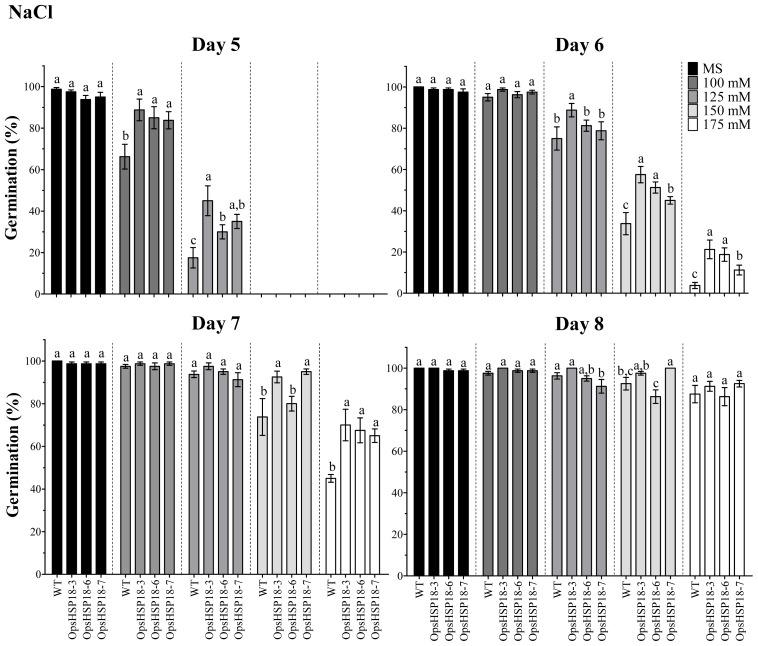
Percentage of seed germination of *A. thaliana* Col-0 and *35S::OpsHSP18*-3, -6, and -7 transgenic lines under salt stress treatments. Seeds were germinated with 0, 100, 125, 150, and 175 mM of NaCl and seed germination percentages were evaluated at 5, 6, 7, and 8 days of treatment. Bars represent the means ± SE (*n* = 20) of four replicates. Different letters indicate significant differences between the WT and the three transgenic lines. One-way ANOVA was used to analyze the data (*p* ≤ 0.05) and differences among treatments were explored through Tukey tests.

**Figure 4:**
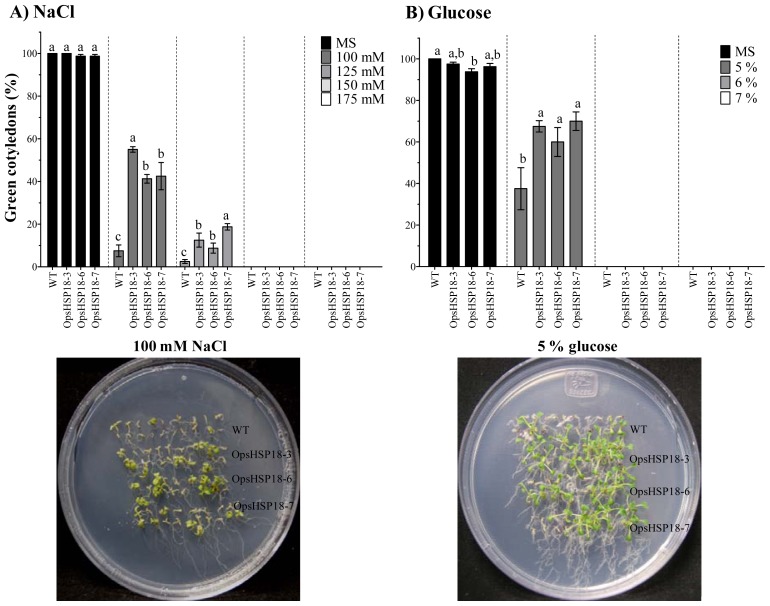
Percentage of green cotyledons in *A. thaliana* Col-0 and *35S::OpsHSP18*-3, -6, and -7 transgenic lines, under salt and osmotic stress treatments. Green cotyledons percentage was evaluated 21 days after seed germination on conditions of 0, 100, 125, 150, and 175 mM NaCl (**A**) and 0%, 5%, 6%, and 7% of glucose (**B**). Images of green cotyledons are shown for treatments of 100 mM NaCl, and 5% glucose at 21 days. Bars represent the means ± SE (*n* = 20) of four replicates. Different letters indicate significant differences between the WT and the three transgenic lines. One-way ANOVA was used to analyze the data (*p* ≤ 0.05) and differences among treatments were explored through Tukey tests.

To determine whether *OpsHSP18* gene plays a role in tolerance to salt stress, seeds of the WT and the over-expressing lines (*35S::OpsHSP18*-3, -6, and -7) were germinated on MS medium containing 0, 100, 125, 150, and 175 mM NaCl. The *35S::OpsHSP18*-3, -6, and -7 transgenic lines exhibited a higher percentage of germination than the WT at all salt concentrations tested (Figure 3). In particular, 6 days after germination on 150 mM NaCl there was a significant increase in the germination rate of *35S::OpsHSP18*-3, -6, and -7 over-expressing lines showing 57.50%, 51.25%, and 45.00%, respectively, compared with 33.75% of the WT (Figure 3). Instead, no significant differences in germination rate between the transgenic lines and the WT were observed under control conditions (Figure 3). We also analyzed the percentage of green cotyledons 21 days after growth under salt stress (Figure 4A). The seedlings of the over-expressing lines (*35S::OpsHSP18*-3, -6, and -7) presented 55.00%, 41.25%, and 42.50% of green cotyledons, respectively, in comparison with 7.50% of green cotyledons in the WT at 100 mM NaCl (Figure 4A). As observed in the figure, transgenic seedlings were clearly more tolerant than the WT, showing enhanced growth under stress conditions.

**Figure 5:**
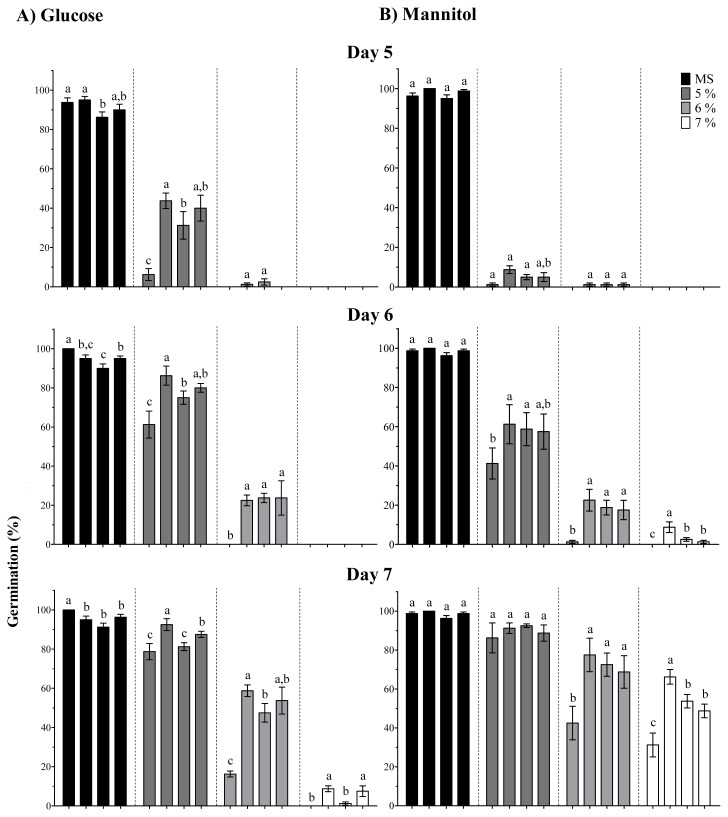

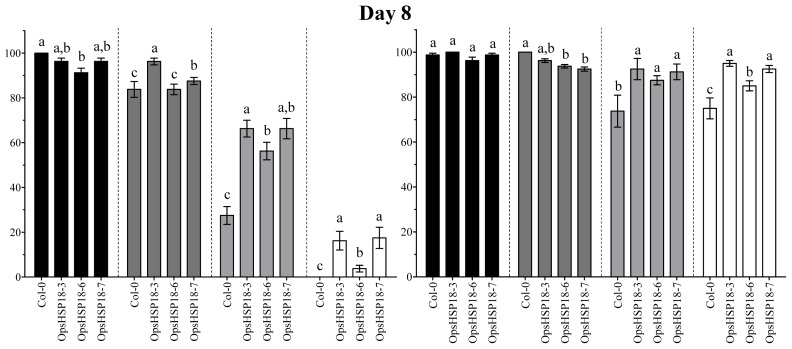
Percentage of seed germination of *A. thaliana* Col-0 and *35S::OpsHSP18*-3, -6, and -7 transgenic lines under osmotic stress treatments. Seeds were treated with 0%, 5%, 6%, and 7% of glucose (**A**) and mannitol (**B**). These concentrations are equivalent to 0, 277, 333, 388 mM of glucose and 0, 274, 329, 383 mM of mannitol. Seed germination percentage was scored at 5, 6, 7, and 8 days of treatments. Bars represent the means ± SE (*n* = 20) of four replicates. Different letters indicate significant differences between the WT and the three transgenic lines. One-way ANOVA was used to analyze the data (*p* ≤ 0.05) and differences among treatments were explored through Tukey tests.

Then seeds of *35S::OpsHSP18*-3, -6, and -7 over-expressing lines and of the WT were germinated in the presence of several glucose or mannitol concentrations (0%, 5%, 6%, and 7%). In Figure 5, the percentage of germination at 5 to 8 days of osmotic stress treatment is shown. In a similar way as described in the salt stress experiments, the over-expression of the *OpsHSP18* gene had a positive effect on Arabidopsis seed germination under sugar treatments. This effect was even more evident at the highest concentrations of glucose and mannitol, where all *35S::OpsHSP18* transgenic lines displayed accelerated seed germination as compared with the WT (Figure 5). The percentage of green cotyledons was estimated 21 days after germination under osmotic stress. It was noticed that all transgenic lines had a higher percentage of green cotyledons (*35S::OpsHSP18*-3: 67.50%, *35S::OpsHSP18*-6: 60.00%, and *35S::OpsHSP18*-7: 70.00%) than the WT (37.50%) in treatments with 5% of glucose (Figure 4B). As mentioned above for salt treatment, growth of transgenic lines under glucose was more vigorous than the WT. In the case of 5% of mannitol, the development of the green cotyledons was arrested in both WT and over-expressing lines (*35S::OpsHSP18*-3, -6, and -7; data not shown).

**Figure 6:**
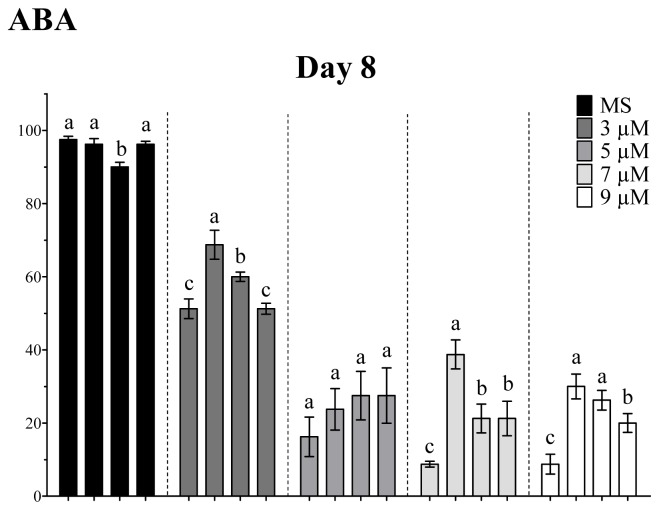

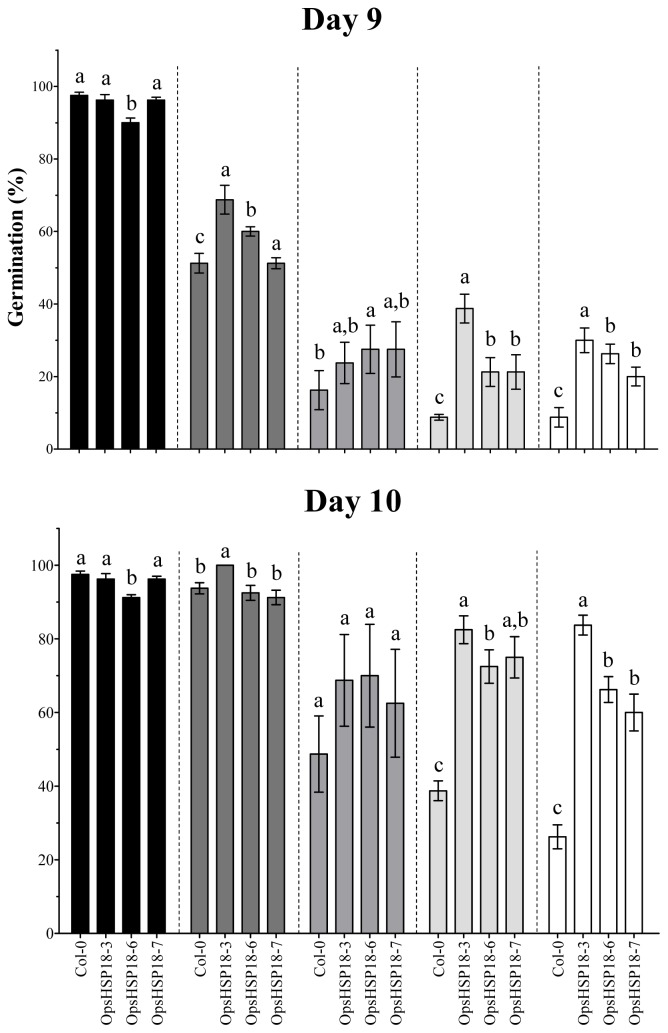
Effect of ABA on the percentage of seed germination of *A. thaliana* Col-0 and *35S::OpsHSP18*-3, -6, and -7 transgenic lines. Abscisic acid (ABA) concentrations were of 0, 3, 5, 7, and 9 μM. Seed germination percentage was evaluated at 8, 9, and 10 days of treatment. Bars represent the means ± SE (*n* = 20) of four replicates. Different letters indicate significant differences between the WT and the three transgenic lines. One-way ANOVA was used to analyze data (*p* ≤ 0.05) and differences among treatments were explored through Tukey tests.

Remarkably, Arabidopsis seeds over-expressing *OpsHSP18* gene show reduced sensibility to ABA (Figure 6). In particular, when the seeds were germinated in the presence of 9 μM of ABA, the transgenic lines achieved more than 60.00% germination as compared to 30.00% of the WT at 10 days of treatment. On the other hand, at all concentrations (3, 5, 7, and 9 μM) of ABA tested, the development of the green cotyledons was arrested in both WT and over-expressing lines (*35S::OpsHSP18*-3, -6, and -7; data not shown).

### 2.3. The Over-Expression of the *OpsHSP18* Gene Allows *A. thaliana* Transgenic Plants Recover from Abiotic Stress

**Figure 7:**
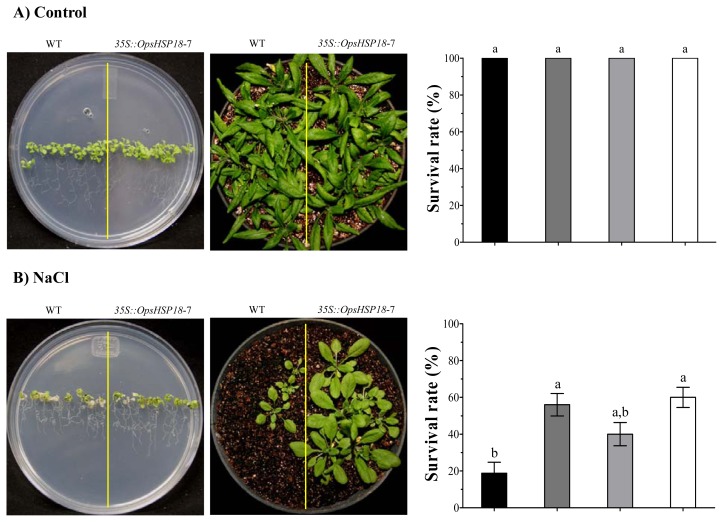

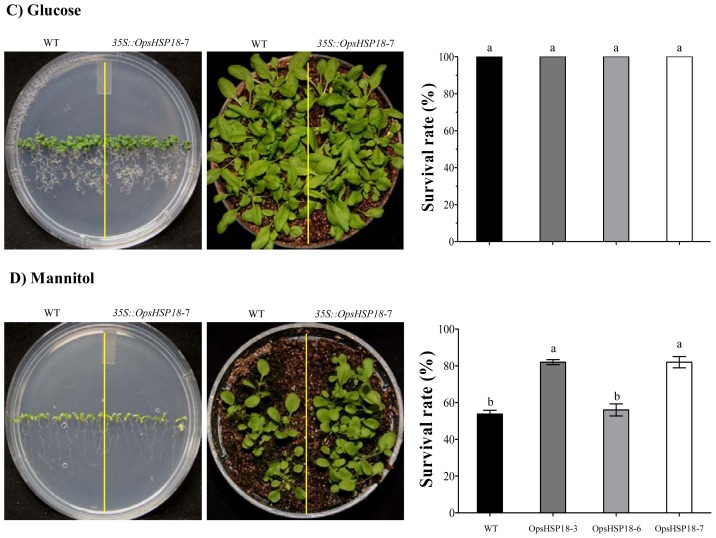
Survival rate of *Arabidopsis thaliana* Col-0 and *35S::OpsHSP18*-3, -6, and -7 transgenic lines after salt (NaCl) and osmotic (glucose and mannitol) treatments. Ten day-old seedlings were grown under: **A**) control conditions in MS medium for 21 days and, transferred to pots for 21 days; **B**) in Murashige and Skoog (MS) medium with 150 mM of NaCl for 14 days; **C**) in MS medium with 7% (388 mM) glucose, and **D**) with 5% (274 mM) mannitol during 21 days. After these stress periods, plants were transferred to pots, and survival rates of seedlings were evaluated at 21 days. Bars represent the means ± SE (*n* = 10) of four replicates. Different letters indicate significant differences between the WT and the three transgenic lines. One-way ANOVA was used to analyze data (*p* ≤ 0.05) and differences among treatments were explored through Tukey tests.

To further characterize the response of the over-expressing lines (*35S::OpsHSP18*-3, -6, and -7) to abiotic stress, salt and osmotic stress treatments were conducted at the seedling stage. Ten day-old seedlings (WT and over-expressing lines) were grown on MS medium containing 150 mM NaCl for 14 days (Figure 7B). When the seedlings of the WT and the transgenic lines were grown under control conditions no significant differences were observed (Figure 7A). However, under salt stress, leaves of the WT seedlings were more affected than those of the transgenic lines showing more symptoms of chlorosis (Figure 7B and Figure S3B). After salt stress treatment, plants were allowed to recover for 21 days in pots, and the percentage of survival was recorded (Figure 7B). We observed that the over-expressing lines *35S::OpsHSP18*-3 and -7 exhibited three fold higher survival rates than control plants, and the over-expressing line *35S::OpsHSP18*-6 achieved two fold (Figure 7B). The same behavior was observed with 5% of mannitol, in which 10 day-old seedlings were grown on MS medium with the sugar for 21 days, and then plants were let to recover in pots for 21 days. Under this condition, the over-expressing lines *35S::OpsHSP18*-3 and -7 showed the highest survival rates (Figure 7D). In contrast, when the seedlings were subjected to 7% of glucose for 21 days in MS medium and recovered, there were no differences between the over-expressing lines (*35S::OpsHSP18*-3, -6, and -7) and the control (Figure 7C). Our results suggest that over-expression of *OpsHSP18* gene in Arabidopsis plays an important role in germination under salt and osmotic stress, and also at the seedling stage, as part of the mechanisms of tolerance to abiotic stress conditions.

### 2.4. Expression of Stress Related Genes in the *A. thaliana OpsHSP18* Transgenic Plants

**Figure 8:**
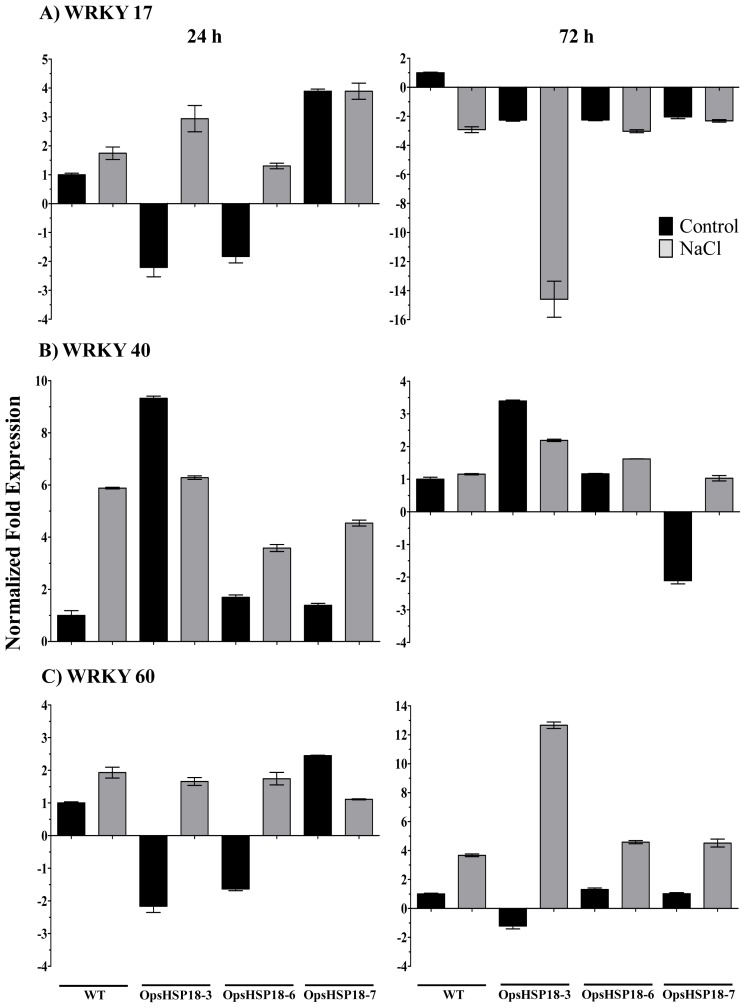
Expression levels of *WRKY* genes in *Arabidopsis thaliana* Col-0 and *35S::OpsHSP18*-3, -6, and -7 transgenic lines under salt stress. Expression levels of *WRKY*-17, *WRKY*-40, and *WRKY*-60 genes were analyzed by qRT-PCR. Data were expressed as relative mRNA level compared with the WT plants without salt treatment, and were calculated after normalization to the Arabidopsis *Actin8* gene using the comparative threshold method. Control conditions (dark bars) and salt stress treatments (gray bars) at 24 and 72 h are shown for Col-0 and *35S::OpsHSP18*-3, -6, and -7 transgenic lines. Error bars were presented to indicate the standard error of the mean.

There is evidence that WRKY transcription factors are involved in plant responses to abiotic stress. We selected the *WRKY*-17, *WRKY*-40, and *WRKY*-60 transcription factors that are known to be induced by salt stress, to evaluate their expression levels by qRT-PCR. For this purpose, 10 day-old seedlings (WT and over-expressing lines) were grown on MS medium supplemented with 0 and 150 mM NaCl during 24 and 72 h. As shown in Figure 8, *WRKY*-17 transcript was induced at 24 h of salt stress (gray bars) in both WT and transgenic lines, and repressed after 72 h (Figure 8A). *WRKY*-17 induction was higher in the transgenic lines *35S::OpsHSP18*-3 and -6 under salt stress at 24 h (Figure 8A). Regarding *WRKY*-40, a notable induction was observed in WT plants subjected to salt stress, under this condition transgenic lines were less induced than the WT. The *35S::OpsHSP18*-3 line was repressed at 24 and 72 h under salt stress (gray bars) in comparison to the condition without stress (black bars). At 72 h, transgenic lines maintained the same expression pattern shown at 24 h under salt stress, while the WT reached control levels (Figure 8B). On the other hand, *WRKY*-60 transcript levels were induced at both 24 and 72 h of salt stress in both control and transgenic lines, the exception was the *35S::OpsHSP18*-7 over-expressing line which was repressed at 24 h of stress (Figure 8C).

## 3. Discussion

Plants are sessile organisms, which are daily exposed to unfavorable conditions such as heat, cold, salt, drought, among others, which limit their growth and development. Processes such as seed germination, seedling growth and vigor, vegetative growth, flowering and fruit set are adversely affected by high salt concentration, ultimately causing diminished economic yield and also reduced quality of production [29]. Thus, plants have developed mechanisms such as the synthesis of heat shock proteins to adapt to those complex environments.

Recently, our research group generated a cDNA library from *Opuntia streptacantha* plants under abiotic stress conditions [24]. Among 329 unigenes of this cDNA library, 9% (29 unigenes) were involved in stress responsiveness, such as the heat shock proteins: HSP90, HSP70, HSP40, and sHSP. sHSPs are associated to the nuclei, cytoskeleton, and membranes, and they bind partially to denatured proteins, avoiding irreversible protein aggregation during stress conditions [16].

In this study, the small heat shock protein (OpsHSP18) was selected for functional characterization. Cactus pear OpsHSP18 protein displays characteristic features of the sHSP family, such as the α-crystalline domain, which has been reported to be involved in substrate binding and, in the interaction between the subunits of oligomeric complexes formed by sHSPs [30]. This domain is flanked by an *N*-terminal region of variable size and sequence, which has been proposed to have a role in unfolded substrate binding and specificity, and finally the *C*-terminal region which has been suggested to confer stability to the oligomeric structure [14].

In the *Arabidopsis thaliana* genome, 19 genes encoding sHSP proteins have been described and classified into six groups, the first three groups of sHSP belong to proteins that are located in the cytoplasm and nucleus (classes CI, CII, and CIII), and those located in mitochondria, plastids and endoplasmic reticulum (classes M, P, and ER, respectively) forming the remaining groups [18]. Thus, based on the alignments and phylogenetic analyzes, the *O. streptacantha* OpsHSP18 protein was grouped within class CI sHSP. Regarding Arabidopsis, it was shown that the AtHSP17.4, AtHSP18.1, and AtHSP17.6C proteins presented the highest percentage of identity with the cactus pear OpsHSP18 protein. Particularly, this conservation was found in the α-crystalline domain and the *C*-terminal region, while the *N*-terminal region of these sHSPs was the most variable.

Several studies have demonstrated that sHSPs play certain roles in plant development such as embryogenesis, seed maturation, seed imbibition and germination, pollen development and fruit maturation under normal and stress conditions [12,26,31–34].

In order to gain insight into the function of the *OpsHSP18* gene under abiotic stress, *A. thaliana* over-expressing lines were generated. When seed germination experiments were conducted under salt and osmotic stress, all three over-expressing lines exhibited higher germination percentages than the WT. Furthermore, over-expression of the cactus pear *OpsHSP18* gene shows a positive effect on seedling development under stress, in which, Arabidopsis seedlings displayed a greater number of green cotyledons under salt and glucose conditions. Thus, our data suggest that over-expression of *OpsHSP18* gene in *A. thaliana* might influence seed vigor under stressful germination conditions.

In general, plant reproductive organs are more sensitive to stress conditions than vegetative organs, so that class CI sHSP can facilitate either acquisition of desiccation tolerance, or they function in the rehydration mechanism of embryos during seed germination [33,35]. Wehmeyer in 1996 [31] found that AtHSP17.4 and AtHSP17.6C proteins that belong to the class CI sHSP of Arabidopsis are expressed in seed maturation. In addition, the expression of *AtHSP17.4*, *AtHSP17.6C* and *AtHSP18.1* appear to be induced under heat stress conditions at temperatures of 35 or 40 °C for 2 h [36], and at 37 °C for 90 min [37]. AtHSP17.6 protein encodes a class CII sHSP from *A. thaliana*, it was detected in mid-maturation, being the transcript more abundant in dry seeds, and a rapid decline was reported 6 days after germination [38]. On the other hand, high levels of *AtHSP17.6A* transcripts accumulated in siliques along with acquisition of desiccation tolerance by seeds. *AtHSP17.6* (class CII) transcripts were less abundant than *AtHSP17.6A* transcripts in seeds, but a strong and transient induction of *AtHSP17.6* transcript (CII) was observed after imbibition [38]. This suggests a putative role of AtHSP17.6 (CII) in protecting proteins during rehydration, and a divergence in function between the two cytosolic AtHSP17.6 proteins.

Recently, the characterization of *NnHSP17.5* gene from *Nelumbo nucifera*, which encodes a cytoplasmic class CII sHSP was reported [34]. It was found that during seed germination the expression levels of *NnHSP17.5* declined rapidly within 3 d after imbibition, suggesting a role in early stages of seed germination. This gene was also found to be strongly regulated under heat and oxidative stress [34].

On the other hand, we found that the over-expression of *OpsHSP18* gene in Arabidopsis increases significantly the germination rate under ABA treatments compared with WT. It has been suggested that ABI3 transcription factor may be necessary to induce several sHSP during seed development [31,32]. Our data show an ABA insensitive phenotype, since the over-expressing lines are less affected in the germination process under ABA treatments than the WT. This observation opens new questions about the role of sHSP and ABA signaling.

In addition, the over-expression of *OpsHSP18* gene confers tolerance to salt and osmotic (mannitol) stress, increasing the survival rate of Arabidopsis transgenic lines after stress recovery. Differences in expression levels of the over-expressing lines were observed, in which a correlation between higher expression levels of lines *35S::OpsHSP18*-3 and -7, and their tolerance to abiotic stress was obtained.

Different studies have shown that over-expression of *sHSPs* confer tolerance to stress conditions. In transgenic rice, the over-expression of the *sHSP17.7* gene confers tolerance to heat and UV-B [39], and drought stress [40]. *NnHSP17.5* over-expression in Arabidopsis transgenic plants showed an increase in the thermotolerance compared with WT plants at 40 °C for 60 or 75 min. The authors suggested that *NnHSP17.5* might play a protective role of proteins under stress conditions, such as superoxide dismutase (SOD), since SOD enzyme activity was found to be higher in transgenic lines than in WT plants [34]. Furthermore, over-expression of *NnHSP17.5* in Arabidopsis transgenic lines confers tolerance to heat, salinity, drought, and osmotic stress [34].

Finally, transcription factors play an important role in abiotic stress tolerance. In the last few years, changes in expression levels of some *WRKY* transcription factors (*WRKY*-17, -18, -25, -33, -40, and -60) have been described under salt stress [41,42]. In addition, some sHSP contain W-boxes on their promoter regions [43], suggesting the transcriptional activation of *sHSP* genes by *WRKY* transcription factors. We observed a notable induction of the transcription factors *WRKY*-17, -40 and -60 in the WT plants after 24 h of under salt stress. However, in the *35S::OpsHSP18* over-expressing lines, the expression level of these transcription factors was altered under stress. In particular, *WRKY*-17 and -60 were more induced in the *35S::OpsHSP18*-3 line than in the WT under stress; perhaps one of the mechanisms that lead stress tolerance in this line during germination under salt stress might be related to *WRKY* expression.

## 4. Experimental Section

### 4.1. Plant Material and Growth Conditions

Seeds of *Arabidopsis thaliana* Columbia ecotype (Col-0) and seeds of the *35S::OpsHSP18* over-expressing lines generated in this study (see below) were sterilized with 20% (*v*/*v*) commercial sodium hypochlorite (6% free chlorine) solution for 5 min, and rinsed five times in sterile distilled water. Aseptic seeds were germinated in plastic Petri dishes containing Murashige and Skoog (MS) 0.5× medium supplemented with 1.4% agar and 1.5% sucrose. Plates were kept at 4 °C for 3 days and then, incubated at 22 ± 2 °C for 10 days in a growth chamber under a 16 h light/8 h dark photoperiod. Plants were grown to maturity in soil pots in a growth chamber at 22 ± 2 °C with a 16 h light/8 h dark photoperiod.

### 4.2. Inverse Polymerase Chain Reaction (IPCR)

Genomic DNA was isolated from *O. streptacantha* seedlings using the DNeasy Plant Mini Kit (Qiagen, Hilden, Germany), according to the manufacturer’s instructions. Then, 0.5 μg of genomic DNA were digested with 20 units of *Eco*RI at 37 °C, in an overnight incubation. The enzyme was then heat inactivated (75 °C for 20 min), and self-ligation of cohesive ends was conducted 16 °C (overnight incubation) with 0.2 units of T4 DNA ligase in a total volume of 100 μL. PCR was performed with 1 μL of the ligation mixture, 1 μL of InvFw*OpsHSP18* 5′-GATATCTCTGGGAACTAAAGGGG-3′ and InvRv*OpsHSP18* 5′-AAAGAAGCTTGGAATTAGCGAC-3′ primers (20 μM each one), 10× Advantage 2 PCR buffer, 50× dNTPs (10 mM each one) and 1 unit of 50× Advantage 2 polymerase mix (Clontech, Mountain View, CA, USA) in a total volume of 50 μL. PCR conditions were as follows: 94 °C for 5 min; 94 °C for 2 min, 58 °C for 2 min, and 72 °C for 3 min (35 cycles); and 72 °C for 10 min. A second amplification was done using 1 μL of the previous PCR reaction. The PCR products were cloned into pCR^®^4-TOPO^®^ vector (Invitrogen, Carlsbad, CA, USA) and sequenced in the Macrogen Company (Seoul, Korea).

### 4.3. Generation of *Arabidopsis thaliana* Over-Expressing Lines

The ORF sequence (486 pb) of the *OpsHSP18* gene was amplified from a cDNA library of *O. streptacantha* cladodes generated under abiotic stress conditions using the primers Fw*OpsHSP18* 5′-ACCATGTCGCTAATTCCAAG-3′ and Rv*OpsHSP18* 5′-CCTTTAGTTCCCAGAGATATC-3′ [24]. The amplified PCR product was cloned into pCR^®^8/GW/TOPO^®^ (Invitrogen, Carlsbad, CA, USA) entry vector, and subcloned into the pMDC32 expression vector by site-specific recombination using Gateway LR Clonase II Enzyme Mix (Invitrogen, Carlsbad, CA, USA). Then, the construct was transformed into *Agrobacterium tumefaciens* GV2260 strain by electroporation and, was introduced into *A. thaliana* Col-0 plants by floral dip method [44]. Transgenic lines carrying the *OpsHSP18* gene (*35S::OpsHSP18*) were selected on MS 0.5× medium containing hygromycin 50 mg/mL. The T2 generation of transgenic plants was transferred into soil pots and grown in growth chambers under controlled conditions to produce seeds. Homozygous transgenic lines (T3) were used for subsequent analysis of seed germination and tolerance assays under abiotic stress conditions.

### 4.4. RNA Isolation, RT-PCR and Real-Time qRT-PCR Analyses

Total RNA was isolated from leaves of *A. thaliana* Col-0 (WT) and *35S::OpsHSP18* transgenic plants, using the Concert™ Plant RNA Reagent (Invitrogen, Carlsbad, CA, USA) according to the manufacturer’s instructions. cDNA synthesis were performed using the SuperScript™ First Strand Synthesis System for reverse transcriptase polymerase chain reaction (RT-PCR; Invitrogen, Carlsbad, CA, USA) according to the manufacturer’s instructions. One microliter of the RT reaction was used as template for PCR assays to amplify the *OpsHSP18* gene using the following primers: Fw*OpsHSP18* 5′-ACCATGTCGCTAATTCCAAG-3′ and Rv*OpsHSP18* 5′-CCTTTAGTTCCCAGAGATATC-3′. As loading control, the *A. thaliana Actin8* (At1g49240) transcript was amplified using the Fw*Act* 5′-GCCAGTGGTCGTACAACCG-3′ and Rv*Act* 5′-CACGACCAGCAAGGTCGAGACG-3′. PCR amplifications were performed in 25 μL reaction mixtures containing 20 mM Tris-HCl (pH 8.4), 50 mM KCl, 3.5 mM MgCl_2_, 200 μM dNTPs, 0.2 μM each primer, 2.5 U Taq polymerase (Invitrogen, Carlsbad, CA, USA) and 1 μL RT reaction as template. The amplifications were carried out with different numbers of cycles to ensure a linear response in the PCR reaction. Amplification conditions for both genes were as follows: 5 min at 94 °C followed by 28–30 cycles (depending on the transcript) of 30 s at 94 °C; 45 s at 58 °C; 45 s at 72 °C; and 5 min at 72 °C. PCR analyses were repeated twice, using 3 biological replicates, obtaining similar results. Regarding real-time RT-PCR, experiments were performed in a 10 μL reaction mixture made up of 5 μL of Power SYBR^®^ Green RT-PCR Mix (2×), 200 nM of each oligonucleotide, 50 ng of RNA template and 0.08 μL of RT Enzyme Mix (125×) for one-step RT-PCR following manufacturer’s suggestions (Applied Biosystems), and using the StepOne Real-Time PCR Detection System and StepOne Software v2.1 (Applied Biosystems). The thermal cycling conditions consisted of 30 min at 48 °C (cDNA synthesis), 10 min at 95 °C (activation of AmpliTaq Gold^®^ DNA polymerase), followed by 40 cycles for PCR cycling of 15 s at 95 °C for denature and anneal/extend of 1 min at 60 °C. For each sample, two biological replicates (*n* = 2) were analyzed with their respective technical replicates. Quantification of *WRKY*-17 (At2g24570), -40 (At1g80840) and -60 (At2g25000) gene expression was based on a cycle threshold value and transcript data were normalized to the *Actin8* (At1g49240) gene values. Relative expression levels of *WRKY*-17, -40, and -60 in WT and the overexpressing lines with or without salt treatments, were calculated based on corresponding levels in WT plants without salt treatment. Absence of contaminant genomic DNA was confirmed by reactions in which no RT Enzyme Mix reverse transcriptase was added, and with primers for the *Actin8* gene which were designed in flanking exons. The primers used were: Fw*WRKY17* 5′-GAGAAATAGAGGGGTTGGTTTTG-3′ and Rv*WRKY17* 5′-CATCATTTTCTTACATGACACCAC-3′ for *WRKY*-17 transcript, Fw*WRKY40* 5′-AAATCAGCCCTCCCAAGAAACG-3′ and Rv*WRKY40* 5′-CTTCACGACAGTCTCTTCTCTCTGC-3′ for *WRKY*-40 transcript, and Fw*WRKY60* 5′-GGTGGGCTTGAACCAGTTGAGG-3′ and Rv*WRKY60* 5′-AATCTCCCGGAAATAGCAGTCG-3′ for *WRKY*-60 transcript.

### 4.5. Germination Assays under Stress and Hormone Treatments

Seeds of *A. thaliana* ecotype Col-0 and of the *35S::OpsHSP18* over-expressing lines (T3) were germinated under different stress conditions and in presence of the phytohormone abscisic acid (ABA). The effect of salt stress on germination was evaluated on MS 0.5× medium supplemented with 0, 100, 125, 150, and 175 mM NaCl. The effect of osmotic stress on germination was assessed on MS 0.5× medium without sucrose and supplemented with glucose (0, 277, 333, and 388 mM) or mannitol (0, 274, 329, and 383 mM). These concentrations were equivalent to 0%, 5%, 6%, and 7% of each sugar [45]. In addition, seeds were germinated in presence of different concentrations of ABA (0, 3, 5, 7, and 9 μM). The germination assays were carried out using 20 seeds of the control (Col-0) and of the transgenic lines (*35S::OpsHSP18*-3, -6, and -7) per treatment. Data are mean ± SE (*n* = 20) from four biological replicates. The seeds were regarded as germinated when the radicle emerged from the seed coat. In addition, green cotyledon number was scored after 21 days of NaCl, glucose, mannitol and ABA treatments.

### 4.6. Estimation of Seedling Survival Rates under Stress Treatments

Seeds of *A. thaliana* Col-0 and of the *35S::OpsHSP18*-3, -6, and -7 transgenic lines (T3 homozygous lines) were germinated *in vitro* in MS 0.5× Petri dishes in a growth chamber (22 ± 2 °C; 16 h light/8 h dark photoperiod). Subsequently, 10 day-old seedlings (ten plants of WT and ten plants of each one of the transgenic lines) were transferred to MS 1× medium supplemented with 0 and 150 mM NaCl for 14 days. In addition, for osmotic stress assays, seedlings were grown on MS 1× plates (without sucrose) containing 388 mM (7%) and 274 mM (5%) of glucose and mannitol, respectively, for 21 days. After these periods of saline and osmotic stress, the seedlings were transferred to pots, grown under controlled conditions (22 ± 2 °C; 16 h light/8 h dark photoperiod) and irrigated every 3 days, to observe post-stress recovery. The survival rate of plants was calculated by counting the number of plants that survived in each pot, after a period of 21 days. Data are mean ± SE (*n* = 10) from five biological replicates. The phenotype of each line was documented photographically.

### 4.7. *In Silico* Analysis of the OpsHSP18 Protein

The molecular weight and the isoelectric point of the OpsHSP18 protein was predicted using the Compute pI/Mw tool of the Expasy proteomics server at the Swiss Institute of Bioinformatics [46]. The search of homologous sequences of the OpsHSP18 protein were conducted using the blast and keyword search tools in the Phytozome database [47] and the BLAST program (BlastN and BlastX) in two databases (GenBank non-redundant (nr) and EST) of the National Center for Biotechnology Information [48]. Putative protein domains were identified using the Motif Scan tool [49] at the Expasy proteomics server. Multiple protein sequence alignments were carried out using the CLUSTAL W and the T-Coffee programs at the European Bioinformatics Institute [50]. The phylogenetic tree was created by Neighbor joining method using the application of the PHYLIP (Phylogeny Inference Package) 3.67 package [28]. For this, the amino acid sequences of the OpsHSP18 protein and the 19 sHSPs of *A. thaliana* [18] were aligned using MUSCLE version 4.0 version of the EBI database using default values. Then, the aligned sequences were subjected to re-sampling with replacement (1000 bootstrap) using the Seqboot program of PHYLIP package and a distance matrix was calculated with Protdist program using the Henikoff/Tillier [51]. Probability Matrix from Blocks (PMB). The resultant matrices were transformed into multiple trees by the Neighbor program and summarized by the program Consense (both programs of the PHYLIP 3.67 package). The majority rule consensus tree was edited with the MEGA version 5.0 program [52].

### 4.8. Statistical Analysis

The data of seed germination and survival experiments of WT and *35S::OpsHSP18*-3, -6, and -7 transgenic lines (T3) were evaluated by One-way ANOVA analysis and Tukey test using GraphPad Software. The data are presented as the mean ± standard error. Differences at *p* ≤ 0.05 were considered significant.

## 5. Conclusions

The enhanced stress tolerance of *35::OpsHSP18* Arabidopsis lines reveals that OpsHSP18 might have an important role in germination and seedling survival under salt and osmotic stress. In addition, the ABA insensibility phenotype of the *35::OpsHSP18* lines reveals its importance in the germination process. Although the mechanism of action of sHSP is not yet clear, the functional characterization of OpsHSP18 provides new insights into abiotic stress responses mediated by chaperons.

## Supplementary Materials
