# Supplementary material for: The Opuntia streptacantha OpsHSP18 Gene Confers Salt and Osmotic Stress Tolerance in Arabidopsis thaliana
Source: Int J Mol Sci. 2012 Aug 15;13(8):10154–75. doi: 10.3390/ijms130810154 (PMC3431851; doi:10.3390/ijms130810154)
Supplement: Supplementary file 1 [file ijms-13-10154-s001.pdf]

Supplementary Material

Figure S1. Nucleotide and deduced amino acid sequence of OpsHSP18. The canonical TATA-box (boxed), the +1 transcription start site (circled), and 5' and 3'-UTR region (underlined) are indicated.

tgataaaatttgcgaacactaataaatggcttagatattaatgtagaggaatgttatagaacgtccacgagaaatcgacaatcatgggactcccgtctgagaagtccg

cacccaaatgaagccattagaagcttgtagaaaagcgtccaaaacaaggcaaatatggtcccccaaaactttattgtttttctagaaaacatctccccaaacccagaa

aaccccagagatctccatctactcc**tataaata**ccccactctcaatccagctactgccatcaaaacagagcaactcaaaaggcatcgatccacaatccaagcttccaa

actcccttcatcaagttcgatcaatccctaaatttcccaacc**ATGTCGCTAATTCCAAGCTTCTTTGGCAGCCGAAAGACCAACGTATTCGACCCATTCTCCCTGGAC**

M S L I P S F F G S R K T N V F D P F S L D

GTTTGGGACCCATTTGAGGGGTTCTTATCCTCGGCCCTTTCCAATGTGCAGCCGAGTTCAGCTGCCCGAGAGACATCGCAGTTTGTGAGTACGAGGGTAGACTGGAAG

V W D P F E G F L S S A L S N V Q P S S A A R E T S Q F V S T R V D W K

GAAACCCCGGAAGCCCACGTGTTCAAGGTGGATCTTCCTGGGGTTAAGAAAGAGGAGGTGAAAGTGGAGGTTGAGGAGGGCAGAATTCTCCAGATCAGTGGCGAGAGA

K E T P E A H V F K V D L P G V K K E E V K V E V E E G R I L Q I S G E

ACCAAAGAGCAGGAGGAGAAGAATGACAAGTGGCACCGTGTGGAGCGTGCAAGTGGCAAGTTCCTCCGGCGATTCCGCCTCCCCGAGAATGCCAAGATGGAGGAGGTG

R T K E Q E E K N D K W H R V E R A S G K F L R R F R L P E N A K M E E V

AAGGCTGCAATGGAGAACGGTGTGCTTACAGTGACTGTTCCTAAGGTTGAAGAGAAGAAGCCTGAGGTGAAGACCATTGATATCTCTGGGAACTAAaggggaggaagt

K A A M E N G V L T V T V P K V E E K K P E V K T I D I S G N *

tgtgaaagagagcaaaatgacgtgaatatggggttgagtctgtgttggcttttgatgcttcaggagattttatgatttgaataaagagtcatatatatttgatgtat

attgtctctgtaatttgtgcgtttgtctgagtgagtacaactttgtttatgtaaaagtgtcgtgttattcattccaaatttgtgttttctctctttcgtgag

Figure S2. Clustal W alignment of the OpsHSP18 protein and other plant Class CI sHSP: *Citrullus lanatus* ClHSP18.1A (GenBank Accession No. ADU55789), *Eucalyptus grandis* EgHSP18 (Phytozome Accession EucgrB02376), *Populus trichocarpa* PtHSP17.8 (GenBank Accession No. Pt_XP002298362), *Vitis vinifera* VvHSP18.2 (GenBank Accession No. XP_002280935), *Medicago sativa* MsHSP18.2 (GenBank Accession No. P27880), *A. lyrata* AlHSP18 (GenBank Accesion No. XP_002864649) and *A. thaliana* AtHSP17.6C (GenBank Accession No. NP_175759). Characteristic domains are indicated with lines: *N*-terminal, α-crystalline and *C*-terminal domains. Two consensus regions I and II (boxed) and a conserved sequence motif, basic-X-Ile/Val-X-Ile/Val (double underlined). Identical residues (asterisk) and conserved amino acid substitutions (dots) are indicated.

N-terminal

```
EgHSP18       -MSLIPSMFGS-RRTDVYDPFSLDIWDPFDGF-FTSSLTNVPAS--ARETSAFANARIDWKETPEAHVFKADVPGLKREEVKVEVEEGRILQIS 89
PtHSP17.8     MASLIPSFFGS-RKTNVFDPFSLDIWDPFEDL-FSSTLANVPAS--TGETSAFVNARIDWKETPEAHVFKADLPGLKKEEVKVEVEEGRILQIS 90
VvHSP18.2     -MALISSVLGGGRRSNIFDPISLDIWDPFEGFPFSTTLANVPNS--ARETSAFVNTRIDWKETPEAHVFKADLPGLKKEEVKVEVEEGRVLKIS 91
ClHSP18.1A    -MALIPTIFGG-RRSNVFDPFSLDVWDPFEGFPFSNSLANLPSS--ARETSAFANTRIDWKETPQAHIFKADLPGIKKEEVKVEVEEGRVLQIS 90
OpsHSP18      -MSLIPSFFGS-RKTNVFDPFSLDVWDPFEGF-LSSALSNVQPSSAARETSQFVSTRVDWKETPEAHVFKVDLPGVKKEEVKVEVEEGRILQIS 91
MsHSP18.2     -MSLIPSFFGG-RRSNVFDPFSLDVWDPFKDFPFNNSALSASFP---RENSAFVSTRVDWKETPEAHVFKADLPGMKKEEVKVEIEDDRVLQIS 89
AlHSP18       -MSLIPSIFGG-RRSNVFDPFSQDVWDPFEGFFTPSSALANAST--ARDVAAFTNARVDWKETPEAHVFKADLPGLKKEEVKVEVEDKNVLLIS 90
AtHSP17.6C    -MSLIPSIFGG-RRTNVFDPFSLDVFDPFEGFLTP-SGLANAP---AMDVAAFTNAKVDWRETPEAHVFKADLPGLRKEEVKVEVEDGNILQIS 88
                :**.:.:*. *:::::**:* *::***..:    :          : : *..:::**:***:**:**.*:**:::******:*: .:* **
```

Consensus Region II

α-crystalline domain **C-terminal**

```
EgHSP18       GERSKEKEEKNDKWHRVERSSGKFLRRFRLPENAKMDQIKASMENGVLTVTVPKEEEKKPEVKSVDISG- 158
PtHSP17.8     GERSKEQEGKNDKWHRIERSSGKFLRRFRLPGNAKMDQVKASMENGVLTVTIPKAEEKKAEVKAIEISG- 159
VvHSP18.2     GERTKEQEEKNDKWHRVERSSGKFLRRFRLPENAKMDEVKATMENGVLTVRVPKEEVKKPEVKAIEISG- 160
ClHSP18.1A    GERSKEHEEKNDKWHRIERSSGKFMRRFRLPENAKAEEVKASMENGVLTVTVPKIEEKKPEVKSIDISG- 159
OpsHSP18      GERTKEQEEKNDKWHRVERASGKFLRRFRLPENAKMEEVKAAMENGVLTVTVPKVEEKKPEVKTIDISGN 161
MsHSP18.2     GERSVEKEDKNDQWHRLERSSGKFMRRFRLPENAKMDQVKAAMENGVLTVTVPKEEVKKPEVKTIDISG- 158
AlHSP18       GERSKENEEKNDKWHRVERASGKFMRRFRLPENAKMEEVKAKMENGVLTVVVPKAPEKKPQVKSIDISAN 160
AtHSP17.6C    GERSNENEEKNDKWHRVERSSGKFTRRFRLPENAKMEEIKASMENGVLSVTVPKVPEKKPEVKSIDISG- 157
              ***: *:* ***:***:**:**** ****** *** :::** ******:* :**   **.:**:::**.
```

Consensus Region I

Figure S3. Survival of seedlings of *Arabidopsis thaliana* Col-0 and *35S::OpsHSP18*-3 and -6 transgenic lines after salt (NaCl) and osmotic (glucose and mannitol) treatments. Ten day-old seedlings were grown under: (**A**) control conditions in MS medium for 21 days and, transferred to pots for 21 days, (**B**) in MS medium with 150 mM of NaCl for 14 days, (**C**) in MS medium with 7% (388 mM) glucose, and (**D**) with 5% (274 mM) mannitol during 21 days. After these stress periods, plants were transferred to pots, and survival rates of seedlings were evaluated at 21 days.

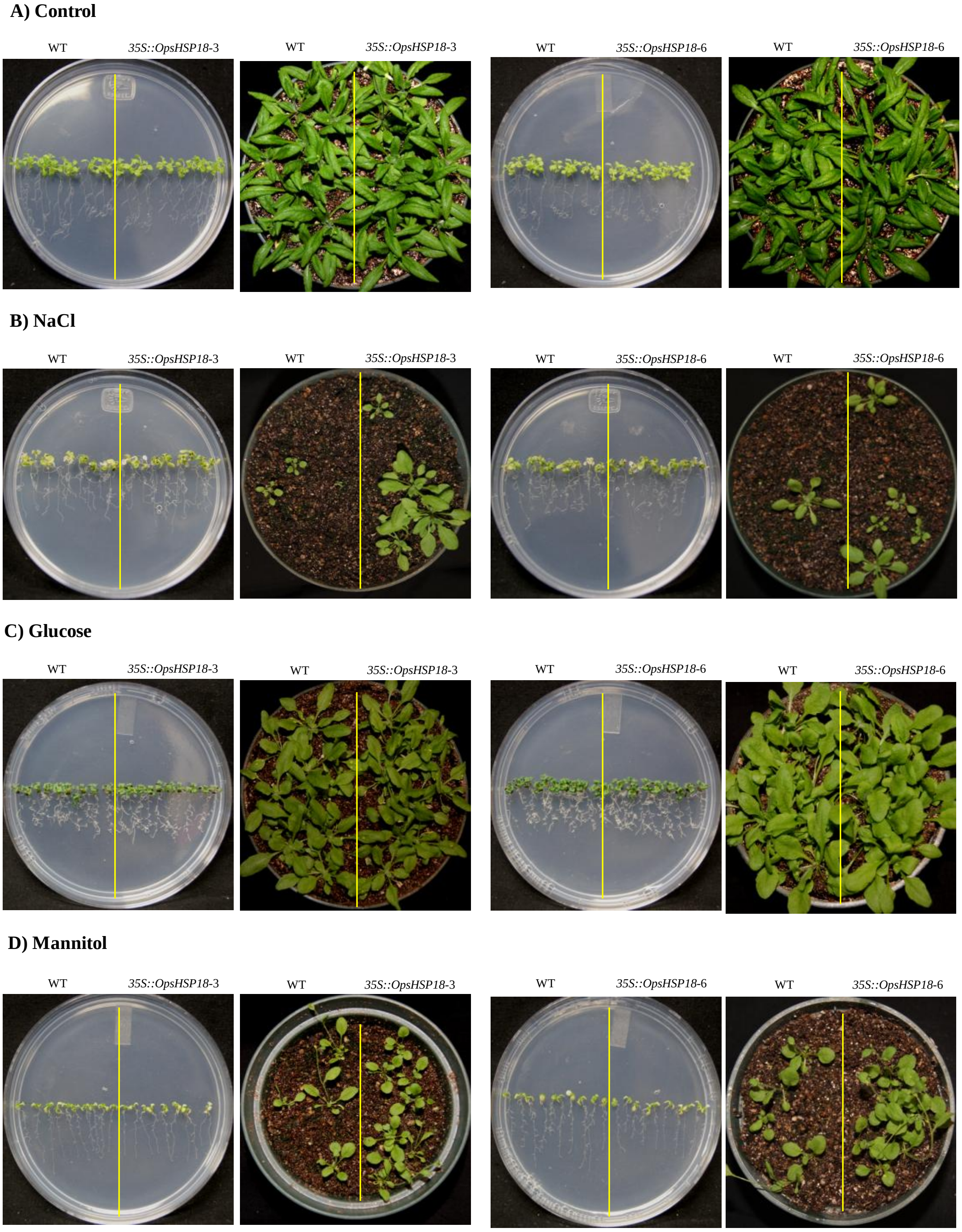

© 2012 by the authors; licensee MDPI, Basel, Switzerland. This article is an open access article distributed under the terms and conditions of the Creative Commons Attribution license (http://creativecommons.org/licenses/by/3.0/).
